# Protein Aggregation and Dysfunction of Autophagy-Lysosomal Pathway: A Vicious Cycle in Lysosomal Storage Diseases

**DOI:** 10.3389/fnmol.2020.00037

**Published:** 2020-03-11

**Authors:** Antonio Monaco, Alessandro Fraldi

**Affiliations:** ^1^Telethon Institute of Genetics and Medicine, Pozzuoli, Italy; ^2^Department of Translational Medicine, University of Naples “Federico II,” Naples, Italy

**Keywords:** lysosome, lysosomal storage disease, autophagy, amyloid aggregation, molecular therapy of neurodegenerative diseases

## Abstract

Many neurodegenerative conditions are characterized by the deposition of protein aggregates (mainly amyloid-like) in the central nervous system (CNS). In post-mitotic CNS cells protein aggregation causes cytotoxicity by interfering with various cellular functions. Mutations in different genes may directly cause protein aggregation. However, genetic factors together with aging may contribute to the onset of protein aggregation also by affecting cellular degradative functions, in particular the autophagy-lysosomal pathway (ALP). Increasing body of evidence show that ALP dysfunction and protein aggregation are functionally interconnected and induce each other during neurodegenerative processes. We will summarize the findings supporting these concepts by focusing on lysosomal storage diseases (LSDs), a class of metabolic inherited conditions characterized by global lysosomal dysfunction and often associated to a severe neurodegenerative course. We propose a model by which the inherited lysosomal defects initiate aggregate-prone protein deposition, which, in turns, worsen ALP degradation function, thus generating a vicious cycle, which boost neurodegenerative cascades.

## Protein Aggregation in Neurodegeneration Diseases

A hallmark of many neurodegenerative diseases is the progressive formation of insoluble protein aggregates that, in most cases, are composed by amyloidogenic proteins (Chiti and Dobson, 2006). Indeed, under different stress conditions, several intrinsically disordered proteins (normally soluble) misfold and undergo structural changes and self-assembly that ultimately lead to their aggregation into insoluble deposits, referred to as amyloids (Dobson, 2003). Amyloid deposits are characterized by a fibrillar morphology and a cross-β structure, whereby intermolecular main-chain hydrogen bonding acts as one major stabilizing interaction (Chiti and Dobson, 2006). Although in some neurodegenerations (e.g., in the polyglutamine diseases; see below) aggregation *per se* could be not the cause of the observed neurotoxicity, generally, amyloid aggregation represents a therapeutic target for neurological conditions since it can cause cytotoxicity either by directly interfering with various cellular functions or because the aggregates sequester other proteins, which play essential cellular functions (Ciechanover and Kwon, 2015; Gallardo et al., 2016). Nevertheless, the mechanisms underlying neurotoxicity driven by amyloid deposition are not completely understood.

Amyloid deposits found in neurodegenerative diseases are often characterized by one main component; however, in some neurodegenerative conditions several amyloidogenic proteins may contribute to amyloid deposition (Table 1). Alzheimer’s disease (AD), the most common neurodegenerative disorder is characterized by deposition of amyloid plaques, whose main component is the amyloid-beta (Aβ) protein (Goedert and Spillantini, 2006). α-Synuclein accumulation and aggregation within Lewy bodies and neurites of the CNS in the form of amyloid fibrils plays a central role in the pathophysiology of Parkinson’s disease (PD) and in a subset of neurodegenerative conditions known as dementias with Lewy bodies (Spillantini et al., 1997). Polyglutamine (polyQ) expansions in unrelated proteins and consequent intracellular accumulation of the mutant protein in inclusion bodies is the underlying cause of a number of inherited rare neurodegenerative disorders, including Huntington’s disease (HD) (polyQ expansion in the huntingtin protein), spinal and bulbar muscular atrophy (SBMA) (polyQ expansion in the androgen receptor protein), and some forms of spinocerebellar ataxias (polyQ expansion in ataxin protein) (Perutz, 1999). Neurofibrillary tangles, which consists of fibrillar aggregates of hyperphosphorylated tau protein, are commonly seen in aging and AD brain and are correlated with decline of brain functions in these conditions (Goedert and Spillantini, 2006). Frontotemporal dementia (FTD), another neuropathy with protein aggregation has also been associated with toxic intracellular aggregates of hyperphosphorylated tau (Lee et al., 2001). Interestingly, some forms of FTD are negative for tau inclusions, while are positive for inclusions containing misfolded TAR DNA-binding protein 43 (TDP-43) (Kwong et al., 2007). TDP-43 inclusions are also found in the amyotrophic lateral sclerosis (ALS), the most common forms of motor neuron disease (Kwong et al., 2007). Aggregate containing the carboxy terminal fragment of APP (APP-βCTF) have been found in Down Syndrome, a neurodevelopmental disorder with pathological features common to the early onset forms of AD (Ying et al., 2019). Amyloid aggregates containing misfolded prion protein (PrP) cause the so-called prion diseases, a group of rare neurodegenerative conditions characterized by the capability of misfolded PrP to transmit their pathological shape onto normal variants of the same protein (Aguzzi and Heikenwalder, 2006). The accumulation of different unrelated misfolded proteins, including the neuronal intermediate filaments (NFs), is a hallmark of the Charcot–Marie–Tooth disease, the most common inherited neuromuscular disease (Theocharopoulou and Vlamos, 2015; Didonna and Opal, 2019). Aggregates containing NFs are frequently observed also in other motor neuron diseases. Lysosomal storage diseases (LSDs) are a group of metabolic diseases caused by inherited defects in lysosomal or non-lysosomal proteins leading to lysosomal storage and global dysfunction often associated with neurodegeneration (Schultz et al., 2011; Platt et al., 2012, 2018). In several LSDs the primary storage caused by the specific inherited lysosomal defect is associated to the deposition of amyloidogenic proteins. Accumulation of α-synuclein has been shown to trigger neurotoxicity through aggregation-dependent mechanisms in Gaucher disease, a severe neurological LSD belonging to the sphingolipidoses, a family of LSDs characterized by primary lipid storage (Mazzulli et al., 2011). α-Synuclein aggregation and neurofibrillary tangles have been observed also in other sphingolipidoses, such as the Niemann–Pick and the Krabbe diseases (Suzuki et al., 1995; Saito et al., 2004; Smith et al., 2014). Accumulation and amyloidogenic processing of an oversialylated APP in lysosomes, and extracellular release of Aβ peptides have been observed in a mouse model of sialidosis, an LSDs caused by the deficiency of the lysosomal sialidase NEU1 (Annunziata et al., 2013). Accumulation of APP-βCTF was found in GM1 gangliosidosis, an LSD characterized by primary lysosomal storage of GM1 ganglioside in neurons (Zha et al., 2004). Mucopolysaccharidoses (MPS) are a family of LSDs with primary storage of glycosaminoglycans (GAGs) due to the deficiency of lysosomal enzymes required for GAG stepwise degradation (Clarke, 2008). Amyloid aggregation has been observed in different types of MPSs, including the MPS type IIIA, one of the most common and severe form of neurodegenerative LSD (Ginsberg et al., 1999; Hamano et al., 2008; Ohmi et al., 2009; Martins et al., 2015; Beard et al., 2017; Sambri et al., 2017; Monaco et al., 2020). In particular, by studying a mouse model of MPS-IIIA, we have shown that brain deposition of α-synuclein together with other amyloidogenic proteins including tau, Aβ, and PrP trigger neurodegenerative processes by both loss-of-function (LOF) (Sambri et al., 2017) and gain of toxic function mechanisms (Monaco et al., 2020) (see next sections for further discussion).

**TABLE 1:** Protein aggregation in neurodegenerative diseases.

**Neurodegenerative disease**	**Aggregating protein/s**
Alzheimer’s disease	Aβ, tau
Parkinson’s disease	α-syn, tau
Dementia with Lewy bodies (DLB)	α-syn
PolyQ expansion diseases (Huntington’s, others)	PolyQ expanded proteins (PolyQ htt, others)
Frontotemporal dementia (FTD)	TDP-43, tau
Amyotrophic lateral sclerosis (ALS)	TDP-43
Prion diseases	PrP
Charcot–Marie–Tooth disease	NFs and other misfolded proteins
Down syndrome	APP-β-CTF
Lysosomal storage diseases	
Mucopolysaccharidoses	Multiple amyloid proteins (α-syn, Aβ, tau, PrP)
Gaucher disease	α-syn
Krabbe, NPC-1	α-syn, tau
GM1 gangliosidoses	APP- β-CTF
Sialidosis	APP, Aβ

## Factors Determining Protein Aggregation

**FIGURE 1:**
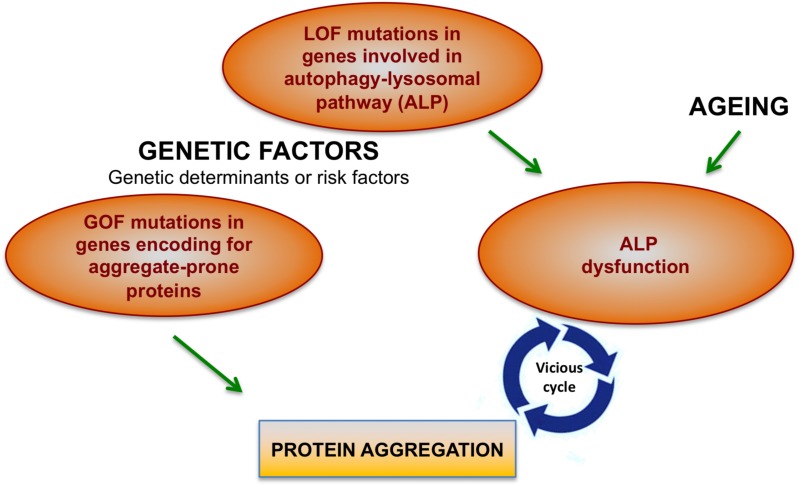
Factors contributing to protein aggregation in neurodegenerative conditions. Mutations in different genes may directly cause protein aggregation. However, genetic factors together with aging may contribute to the onset of protein aggregation also by affecting cellular degradative functions, in particular the autophagy-lysosomal pathway (ALP). Increasing body of evidence show that ALP dysfunction and protein aggregation are functionally and closely interconnected and induce each other during neurodegenerative processes.

There are two main factors that cause protein aggregation in neurodegenerative diseases: Gain-of-function (GOF) dominant mutations in genes encoding aggregate-prone proteins and the decline of cellular degradation functions, in particular of the autophagy-lysosomal system (Figure 1).

### GOF Mutations in Genes Encoding Aggregate-Prone Proteins

Protein aggregation may be directly caused by dominant GOF mutations in gene encoding aggregate-prone proteins or precursors of aggregate-prone proteins. GOF mutations in the gene encoding huntingtin lead to polyQ expansion and huntingtin aggregation, thus causing HD (Perutz, 1999). In the AD, ∼5% of the forms are Mendelian and are caused by mutations in the genes encoding the amyloid precursor protein (*APP*) and presenilin1/2 (*PSEN1/2*). These genes are directly involved in the “amyloidogenic cascade” by which the APP protein is sequentially cleaved and processed to generate aggregate-prone Aβ peptides (Hardy and Selkoe, 2002). While PSEN1/2 mutations increase the activity of γ-secretase that enhances Aβ peptides production, GOF mutations in *APP* gene increase the generation of Aβ peptides either by making APP a better substrate for its processing or by changing the biophysical properties of the Aβ peptide, thus rendering it more likely to aggregate. Among the rare Mendelian forms of PD a number of cases are associated with dominant GOF mutations in the gene encoding α-synuclein (*SNCA*), which lead to the abnormal aggregation of the protein (Bras et al., 2015). Some GOF mutations in the *PRNP* gene (encoding the PrP protein) produce altered misfolded-prone versions of PrP, thus causing prion disease (Beck et al., 2010). Genetic studies identified GOF mutations in the *MAPT* gene encoding tau protein in some familial cases of the FTD (Rainero et al., 2017). GOF mutations in the *TARDBP* gene (encoding TDP-43) are associated with FTD and ALS (Kwong et al., 2007). In Down syndrome the extra gene copy of *APP* gene (on chromosome 21) leads to increased production of APP-βCTF (Ying et al., 2019). Finally, several GOF mutations in unrelated genes have been found to cause misfolding and accumulation of the corresponding proteins in Charcot–Marie–Tooth disease (Braathen, 2012).

### Decline of the Autophagy-Lysosomal Pathway

In many neurodegenerative conditions protein aggregation may occurs without specific GOF mutations in genes encoding aggregate-prone proteins. In these conditions protein aggregation is associated to the decline of cellular degradative functions, specifically of the autophagy-lysosomal pathway (ALP) (Figure 1). ALP is a major process for degrading intracellular macromolecules and generating energy or building blocks to make other macromolecules. ALP relies on the engulfment of cargos to be degraded (macromolecules or damaged organelles) in double-membrane vesicles (autophagosomes), which, therefore, fuse with endosomes/lysosomes to form autolysosomes, where autophagosome contents are degraded by lysosomal enzymes (Yu et al., 2018). ALP plays a key role in protein homeostasis and in the clearance of protein aggregates (processes that are particularly important in non-dividing neurons). Therefore, ALP dysfunction may determine/contribute to the toxic aggregation in neurodegenerative conditions (Nixon, 2013; Fraldi et al., 2016). Accordingly, mice KO for key ALP components exhibit neuronal accumulation of aggregate-prone proteins and neurodegeneration (Hara et al., 2006; Komatsu et al., 2006). Furthermore, in the case of Aβ aggregation, it has been reported that the functionality of endo-lysosomal recycling trafficking is critical for determining the amyloidogenic cascade (Rajendran and Annaert, 2012; Das et al., 2013) and, therefore, any detrimental effect on the endo-lysosomal transport results in alterations of Aβ production (Nixon, 2017).

Autophagy-lysosomal pathway decline may be caused by genetic factors (LOF – mutations inherited in a dominant or recessive fashion), environmental factors (mainly aging) which are known to impact on degradative capability of cells (Nixon et al., 2008) or by protein aggregation itself (see next paragraph). Therefore, genetic factors trigger protein aggregation in neurodegenerative diseases either directly (GOF mutations in gene encoding aggregate-prone proteins) or indirectly (LOF mutations in ALP genes). Importantly, genetic factors can represent either the genetic determinant or a risk factor that contributes to neurodegenerative conditions (Figure 1).

Lysosomal storage diseases are the paradigm of neurodegenerative diseases associated to ALP dysfunction caused by genetic factors (Fraldi et al., 2016). Indeed, in LSDs LOF mutations in lysosomal hydrolases or in proteins involved in lysosomal biology cause lysosomal storage and global dysfunction associated to the impairment of the autophagy flux (Lieberman et al., 2012; Platt et al., 2012). A number of AD patients carrying LOF mutations in *PSEN1* show lysosomal and autophagic dysfunction (Lee et al., 2010). Lysosome dysfunction in these patients can be explained by two different mechanisms, one involving defects in the lysosomal acidification machinery and the other in lysosomal Ca^+2^ homeostasis (Coen et al., 2012; Lee et al., 2015). Some Mendelian forms of PD are caused by mutations in ALP genes. Mutations in *ATP13A2* encoding a component of the lysosomal acidification machinery (ATPase type 13A2) are associated with lysosomal dysfunction and defective autophagosomes clearance in PD (Ramirez et al., 2006). PD caused by mutations in the *LRRK2* gene showed lysosomal stress and accumulation of abnormal autophagosomes (reviewed in Jin and Klionsky, 2014). PD with mutations in VPS35 is associated to defects in the retrograde transport between endosomes and the trans-Golgi network (Zimprich et al., 2011). Mutations in *PINK* (PTEN-induced putative kinase) or *PARKIN* (PD protein) genes cause PD forms characterized by defective mitophagy (Geisler et al., 2010). Dysfunction of ALP has been associated with specific mutations also in ALS (Song et al., 2012) and in CMT disease (Lee et al., 2012; BasuRay et al., 2013). LOF mutations in the genes whose products are involved in endo-lysosomal function (such as CHMP2B, progranulin, and TMEM106B genes) have been identified as the causative factors in familiar forms of FTD (Rainero et al., 2017).

Mutations in ALP genes may also represent risk/predisposing factors for disease pathogenesis. Interestingly, in PD many ALP gene mutations represent risk factors when they are in heterozygosis, while cause a specific LSD when they are in homozygosis, thus providing a strong genetic evidence linking between PD and LSDs (Shachar et al., 2011; Robak et al., 2017). The most known example of this genetic link is provided by the *GBA* gene encoding for the glucocerebrosidase (GCase), a lysosomal enzyme involved in the degradation of glucosylceramide. When *GBA* is mutated in homozygosis causes the Gaucher’s diseases, while when it is mutated in heterozygosis represents a major risk factor for PD (Sidransky et al., 2009).

## Protein Aggregation May Affect ALP Generating a Vicious Cycle in Neurodegenerative Diseases

As discussed in the previous section, ALP dysfunction may contribute to the toxic aggregation in neurodegenerative diseases. On the other hand, mounting evidence also show that protein aggregation itself may affect ALP, thus generating a vicious cycle, which boost protein aggregation and toxicity (Figure 1). Although mechanisms underlying these processes are still poorly understood, these indirect pathways may explain why ALP became dysfunctional in neurodegenerative conditions caused by GOF mutations in aggregate-prone proteins.

### How Protein Aggregation Affect ALP

Different works have shown that the aggregated forms of α-synuclein can bind the lysosome, thus impairing the chaperone-mediated autophagy, a selective autophagy pathway for degradation of cytosolic proteins (Cuervo et al., 2004; Martinez-Vicente et al., 2008) or inducing lysosomal rupture (Freeman et al., 2013). Moreover, α-synuclein overexpression may compromise ALP by inhibiting autophagy initiation via Rab1a inhibition (Winslow et al., 2010). In addition, α-synuclein toxicity has been reported to be associated with a progressive decline in markers of lysosome function due to cytoplasmic retention of TFEB, a master transcription factor regulating lysosomal biogenesis and function (Settembre et al., 2011; Decressac et al., 2013). Similarly, the polyglutamine-expanded androgen receptor (polyQ-AR) associated to SBMA, interferes with TFEB transactivation, which accounts for autophagic flux defects present in SBMA motor neuron-like cells (Cortes et al., 2014). Abnormal toxic polyQ expansions of htt protein may affect the efficiency of autophagy by inhibiting cargo recognition by autophagosomes (Martinez-Vicente et al., 2010) and/or by inhibiting autophagosome biogenesis and transport (Wong and Holzbaur, 2014; Rui et al., 2015). In Down syndrome increased production of APP-βCTF has been shown to impair lysosomal acidification and function (Ying et al., 2019).

### The Paradigm of Lysosomal Storage Diseases

**FIGURE 2:**
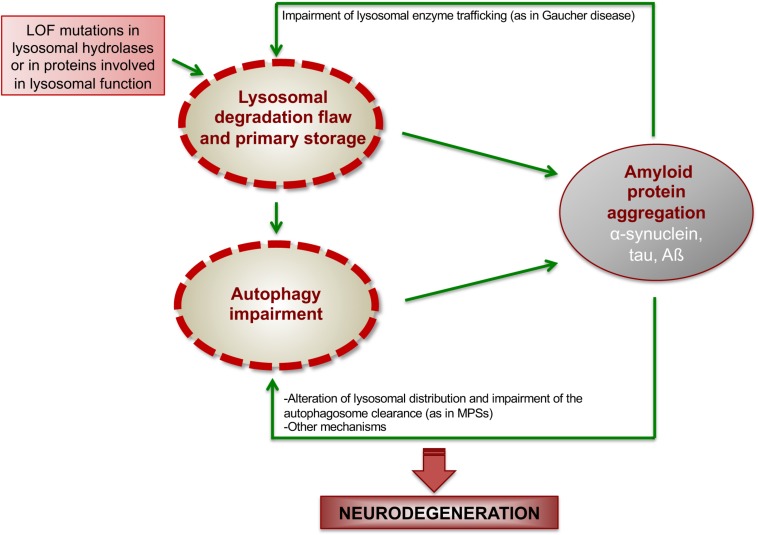
Proposed model showing how ALP dysfunction and protein aggregation generate a vicious cycle in LSDs. In LSDs, the inherited LOF of a specific lysosomal enzyme causes lysosomal degradation flaw and primary storage, which promotes the initial deposition of amyloidogenic proteins. Such amyloid deposition, in turns, worsens lysosomal degradation capability and impairs autophagy function, thus generating a vicious cycle, which boost neurodegenerative cascades.

The interplay between ALP dysfunction, protein aggregation, and neurodegenerative processes is well represented in LSDs (Figure 2). Here, we will provide some key examples of LSDs in which the mechanisms underlying this interconnection have been studied more in depth.

In Gaucher disease lower levels of GCase in the lysosomes lead to the increased accumulation of glucosylceramide, which stabilizes soluble oligomeric α-synuclein intermediates that, in turn, are converted into amyloid fibrils (Mazzulli et al., 2011). The accumulation of α-synuclein inhibits the trafficking of newly synthesized GCase from ER to Golgi, thus reducing the amount of functional GCase in the lysosomes and further amplifying glucosylceramide accumulation. Therefore, the loss of GCase creates a positive feedback loop of reduced lysosomal function and α-synuclein accumulation that ultimately leading to neurodegeneration (Mazzulli et al., 2011).

As discussed above several MPSs show the presence of amyloidogenic protein aggregates in the brain. Nevertheless, the neuropathogenic relevance of these amyloid deposits in the context of MPSs and the underlying mechanisms remain largely unexplored. Recently, we have demonstrated that α-synuclein accumulates as neuronal insoluble aggregates in a mouse model of MPS-IIIA, and showed that this accumulation depletes synaptic α-synuclein, contributing to neurodegeneration by a LOF mechanism (Sambri et al., 2017). Further studies in MPS-IIIA mice have showed that α-synuclein progressively accumulates together with other amyloid proteins, including PrP, tau, and Aβ mostly into the lysosomes of neuronal cell bodies, thus exerting a gain of neurotoxic function by affecting ALP (Monaco et al., 2020). Indeed, inhibiting amyloid aggregation in MPS-IIIA mice by using CLR01, a “molecular tweezer” that acts as a broad-spectrum inhibitor of protein self-assembly (Attar and Bitan, 2014) reduced lysosomal enlargement and re-activates autophagy, thus ameliorating neurodegenerative signs (Monaco et al., 2020). Mechanistically, our preliminary data in MPS-IIIA mouse brain indicate that the build-up of multiple amyloid proteins into the lysosomes of neurons leads to lysosomal clustering in cell body and to the concomitant depletion of the axonal pool of lysosomes, which are critical for autophagosome encountering and clearance. As a consequence, LAMP1-negative autophagosomes massively accumulate in the cell periphery and axons. Therefore, our data suggest a model in which amyloid aggregation impairs the autophagic flux in neurons by disrupting normal lysosomal distribution and, thus preventing lysosomes to encounter and fuse with autophagosomes. Importantly, a similar neuropathogenic link between amyloid deposition and ALP is likely to occur also in other MPSs showing both amyloid deposition (see previous section) and autophagy impairment (Pierzynowska et al., 2019). Furthermore, in addition to the inhibition of lysosomal-mediated clearance of autophagosomes, other mechanisms (such as those discussed above) may contribute to amyloid-induced autophagy impairment in MPSs. Nevertheless, an open question is: if amyloid accumulation accounts for autophagy degradative dysfunction, what triggers initial deposition of amyloid proteins in the MPS brain? In the case of Gaucher disease it has been demonstrated that the primary storage of glucosylceramide stabilizes α-synuclein intermediates, promoting amyloid fibrils deposition (Mazzulli et al., 2011). It is likely that the primary storage of other sphingolipids may trigger amyloid aggregation in other forms of sphingolipidoses where, indeed, amyloid protein deposition has been observed (see the previous section). Similarly, GAGs could initiate and stabilize amyloid deposition in the case of MPSs. Supporting this hypothesis, it has been reported that GAGs provide a scaffold promoting amyloid aggregation (Iannuzzi et al., 2015; Liu et al., 2016).

In summary, findings in Gaucher disease and MPSs suggest a model in which primary lysosomal storage due to the inherited lysosomal deficiency triggers initial amyloid deposition, which, in turns, affect lysosomal functions, including autophagy degradation, thus generating a vicious loop between ALP and amyloid deposition, which boost neurodegeneration (Figure 2).

## Conclusion

The interplay between protein aggregation and ALP dysfunction is crucial in driving neurodegenerative processes in a number of neurological conditions, among which LSDs represent the paradigm. In LSDs genetic factors directly cause the failure in lysosomal degradation function and the storage of undegraded materials into the lysosome. Although the underlying mechanisms are still unclear, it is likely that the primary storage due to the inherited lysosomal defect may promote the initial deposition of amyloidogenic proteins into the lysosomal compartment. Such compartmentalized deposition, in turns, worsens autophagy-lysosomal degradation capability of neurons through different mechanisms that may involve reduced trafficking of lysosomal enzymes to the lysosomes (as in the case of Gaucher diseases), impaired autophagosome clearance (as we demonstrated in MPSs) and, likely, others. These mechanisms generate a vicious loop that boost neurodegenerative processes in LSDs, thus allowing, on the other hand, the possibility to identify new attractive therapeutic targets to treat these severe neurological conditions.

## Data Availability Statement

The datasets generated for this study are available on request to the corresponding author.

## Ethics Statement

The animal studies were conducted in accordance with the guidelines of the Animal Care and Use Committee of TIGEM in Naples and authorized by the Italian Ministry of Health.

## Author Contributions

AF conceived and wrote the manuscript. AM contributed to conceiving the manuscript and co-wrote the manuscript. All authors listed approved it for publication.

## Conflict of Interest

The authors declare that the research was conducted in the absence of any commercial or financial relationships that could be construed as a potential conflict of interest.
